# Comparison of haptic guidance and error amplification robotic trainings for the learning of a timing-based motor task by healthy seniors

**DOI:** 10.3389/fnsys.2015.00052

**Published:** 2015-03-31

**Authors:** Amy E. Bouchard, Hélène Corriveau, Marie-Hélène Milot

**Affiliations:** Centre de Recherche sur le Vieillissement, Université de Sherbrooke, Sherbrooke, QCCanada

**Keywords:** haptic guidance, error amplification, timing, aging, learning

## Abstract

With age, a decline in the temporal aspect of movement is observed such as a longer movement execution time and a decreased timing accuracy. Robotic training can represent an interesting approach to help improve movement timing among the elderly. Two types of robotic training—haptic guidance (HG; demonstrating the correct movement for a better movement planning and improved execution of movement) and error amplification (EA; exaggerating movement errors to have a more rapid and complete learning) have been positively used in young healthy subjects to boost timing accuracy. For healthy seniors, only HG training has been used so far where significant and positive timing gains have been obtained. The goal of the study was to evaluate and compare the impact of both HG and EA robotic trainings on the improvement of seniors’ movement timing. Thirty-two healthy seniors (mean age 68 ± 4 years) learned to play a pinball-like game by triggering a one-degree-of-freedom hand robot at the proper time to make a flipper move and direct a falling ball toward a randomly positioned target. During HG and EA robotic trainings, the subjects’ timing errors were decreased and increased, respectively, based on the subjects’ timing errors in initiating a movement. Results showed that only HG training benefited learning, but the improvement did not generalize to untrained targets. Also, age had no influence on the efficacy of HG robotic training, meaning that the oldest subjects did not benefit more from HG training than the younger senior subjects. Using HG to teach the correct timing of movement seems to be a good strategy to improve motor learning for the elderly as for younger people. However, more studies are needed to assess the long-term impact of HG robotic training on improvement in movement timing.

## Introduction

Movements are made up of spatial (e.g., direction of movement) and temporal (e.g., reaction time and timing of muscle activation) aspects, which can be controlled separately depending on the task that is to be performed (Georgopoulos, 2002). The temporal aspect of movement plays an important role in the accomplishment of many everyday activities like playing tennis (Marchal-Crespo et al., 2013) or using a motorized wheelchair (Marchal Crespo and Reinkensmeyer, 2008). With age, and as compared to younger individuals, a decline in the temporal aspect of movement occurs, translating into a slowing of internalized timing processes, as highlighted by the synchronization-continuation paradigm, and by deficits in temporal predictions for accurate movement production (Carnahan et al., 1996; Wishart et al., 2000; McAuley et al., 2006; van Dijk et al., 2007; Marchal-Crespo et al., 2010; Seidler et al., 2010; Pietschmann et al., 2011; Turgeon and Wing, 2012; Hoogendam et al., 2014), negatively impacting daily activities. Explanations for this age-related timing deficit may be explained by changes in the nervous system (Seidler et al., 2010). For example, a decrease in the nigrostriatal region’s dopamine (DA) functioning, important for motor learning, is observed with age (van Dijk et al., 2007). In fact, van Dijk et al. (2007) found that the availability of the DA transporters (DAT) was negatively correlated (*r* = -0.40) with older individuals’ reaction times; in other words, the slower the reaction time, the higher the deficiency in DAT. Other studies have also found significant relations between decreased functional performance found with aging and decreased fractional anisotropy, a measure of fiber density (Zahr et al., 2009), or decreased volume in the cerebellum, which plays an essential role in movement timing (Raz et al., 2005; Seidler et al., 2010). In addition, it is suggested that elders strategically prioritize the spatial aspects of movement over the temporal aspects, in other words favoring a better control of movements over their speed in order to achieve a required task (Seidler-Dobrin et al., 1998).

Despite these drawbacks, seniors can still learn new motor skills (Voelcker-Rehage, 2008), although often at a significantly slower pace (Marchal-Crespo et al., 2010; Pietschmann et al., 2011). The fact that they have the potential for motor learning is important because the elderly population needs to be able to learn new timing tasks like driving a powered wheelchair, or playing sports such as golf in order to maintain a good quality of life as they age (Carnahan et al., 1996; Wishart et al., 2000; Marchal-Crespo et al., 2010). Yet, few studies have attempted to improve elderly movement timing.

One solution is robotic training (Patton and Mussa-Ivaldi, 2004; Marchal Crespo and Reinkensmeyer, 2008; Marchal-Crespo et al., 2010, 2013; Milot et al., 2010; Luttgen and Heuer, 2013). Two emerging types of robotic training, haptic guidance (HG) and error amplification (EA), are increasingly used as effective training methods for improving movement execution in both healthy persons and those having a pathology (Liu et al., 2006; Patton et al., 2006a,b; Cesqui et al., 2008; Marchal Crespo and Reinkensmeyer, 2008; Marchal-Crespo et al., 2010, 2013, 2014; Milot et al., 2010; Luttgen and Heuer, 2013). HG is based on the principle that guiding the person to make the correct movement would provide the nervous system with additional proprioceptive and somatosensory cues to allow for a better planning of movement and thus reducing timing errors and improving movement execution (Marchal-Crespo et al., 2010; Milot et al., 2010; Luttgen and Heuer, 2013). EA, on the other hand, is based on the theory that error is an essential neural signal for motor adaptation (Thoroughman and Shadmehr, 2000), where the nervous system detects and corrects these errors for future movements (Milot et al., 2010). By artificially increasing movement errors, EA training would then allow a faster and more complete learning (Emken and Reinkensmeyer, 2005). In a previous study, we evaluated and compared the immediate impact of HG and EA robotic trainings on movement timing for 20 young healthy participants, while they played a computerized pinball-like game (Milot et al., 2010). The results showed that both EA and HG training were effective in improving timing accuracy so that it was appropriate for hitting a target with the pinball. Moreover, the effectiveness of the robotic trainings was dependent on the participant’s initial skill level, where less-skilled participants seemed to benefit more from HG and better-skilled participants from EA. The results of our study further supported studies that used HG or EA robotic trainings, where faster execution times (Feygin et al., 2002; Bluteau et al., 2008) and improved timed movement for the task (Marchal Crespo and Reinkensmeyer, 2008; Luttgen and Heuer, 2013; Marchal-Crespo et al., 2013, 2014) were noted following HG or EA training. However, these studies were conducted on young and healthy individuals. Very few studies have compared the effectiveness of both EA and HG to improve movement timing in the elderly, despite there being a negative impact of aging on movement timing. Only one study by Marchal-Crespo et al. (2010) trained elders with HG on a computerized steering task. The authors found that after receiving HG training, elders improved their timing by straightening their wheel faster after having just coming out of turns.

With respect to the benefits of HG and EA robotic training on the performance of timed movement in young healthy individuals, the goal of the study was to directly compare and evaluate the impact of both types of robotic trainings on improving movement timing accuracy in elders. Since learning can still take place with age (Carnahan et al., 1996; Wishart et al., 2000; Marchal-Crespo et al., 2010; Pietschmann et al., 2011) and following our previous results on young healthy individuals (Milot et al., 2010) which showed that timing error can be improved using HG and EA robotic trainings, we expected both EA and HG to be equally effective in improving seniors’ timing errors. However, knowing that there is a decrease in motor skill observed with older age (Hoogendam et al., 2014), we hypothesized that our oldest senior participants would more likely have a poorer task performance and would thus benefit the most from HG, whereas our younger senior participants would not necessarily be bad performers and would therefore benefit more from EA.

## Materials and Methods

### Participants

Thirty-two healthy participants (10 male; 22 female) between the ages of 61 and 75 (mean age 68 ± 4 years) took part in the study. In order to participate in the study, individuals had to meet the following criteria: (1) be at least 60 years of age, (2) be right-handed [*Edinburgh Handedness Inventory* (Oldfield, 1971)], and (3) be able to flex the right wrist by at least 10° without any pain. The exclusion criteria were: having a cognitive impairment as evaluated by the *Montreal Cognitive Assessment* exam [score < 26 on the Version 7.3 (Gluhm et al., 2013)]; having an active neurological (e.g., stroke) or orthopedic (e.g., fracture) problem in the right upper limb; having a visual problem (e.g., a cataract) that was non-corrected and would prevent the proper viewing of a computer screen. This project was approved by the CSSS-IUGS ethics board and all participants signed the consent form.

### Timing Exerciser Orthosis (TEO)

**FIGURE 1:**
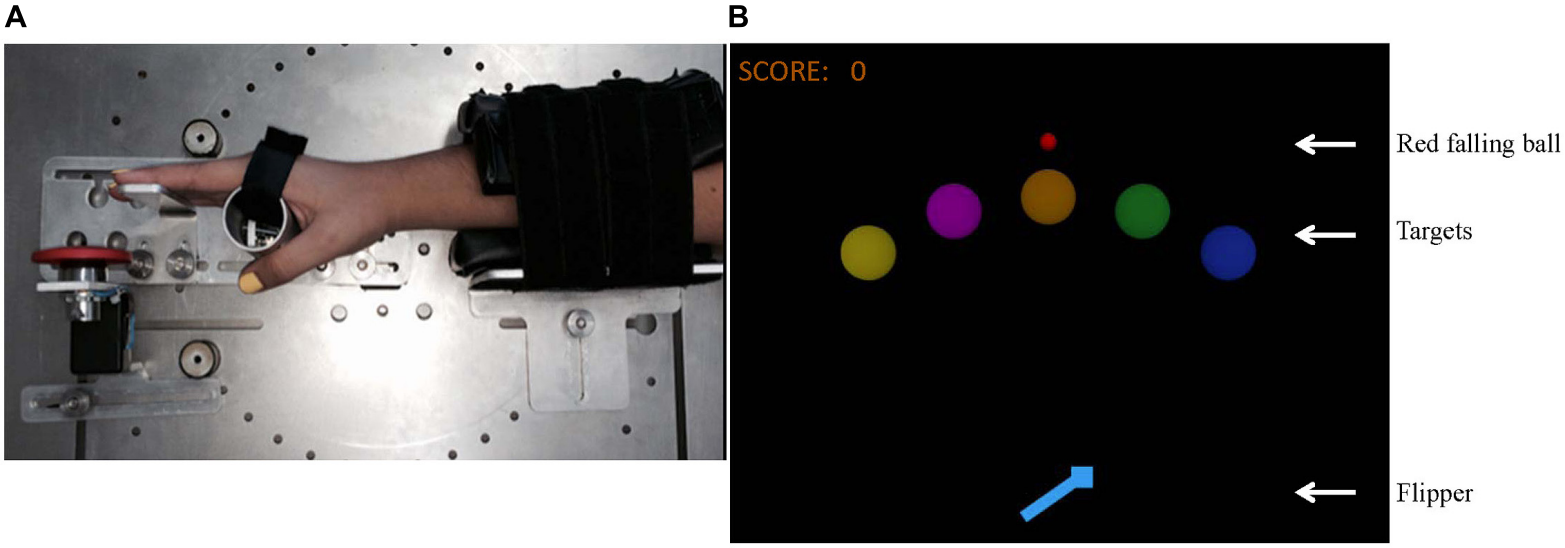
**(A)** The Timing Exerciser Orthosis (TEO) robot; **(B)** Description of the computerized pinball-like game: a red ball appears at the top of the screen and falls toward the virtual flipper. Subjects flex their right wrist to activate TEO. TEO moves the wrist at an angle of 10°, allowing the subject’s fingers to touch the button while rotating the flipper. The ball touches the flipper, and then bounces toward the target.

Timing exerciser orthosis (TEO) is a modified version of TAPPER, which was used in a previous study (Milot et al., 2010). TEO is a one degree of freedom robot that allows 10° of wrist flexion of both the left and right hands. It is mounted on an aluminum frame and is mechanically actuated by a Dynamixel MX-106 servomotor (*Robotis Inc*, USA), sampled at 1,000 Hz for the recording of the servomotor’s torque and position data. A forearm brace also ensures the participant’s comfort and safety. A button, placed on the frame, allows the participants to experience sensory feedback during each trial but pressing the button did not play a role in triggering the sequence of the timing task (see **Figure 1**).

Since the task was time dependent, a photodiode (*BPW21R*, Vishay, Germany), placed at the bottom right corner of the computer screen, was used as a time reference in order to detect the start of the task. This was done by the appearance of a luminous white dot at the bottom right corner of the computer screen, which was read by the photodiode, as a red ball appeared at the top of the computer screen simultaneously. This photodiode and TEO were plugged into a USB-6008 data acquisition card (*National Instruments*, USA), sampled at 5,000 Hz. This acquisition card allowed the synchronization between the photodiode and the servomotor for the timing task.

### Pinball Simulation Game

The pinball-like game is similar to the one used in a previous study [for more details see (Milot et al., 2010)]. In sum, the goal of the game was to hit targets to earn as many points as possible. A total of five colored-targets located at specific positions across the computer screen were randomly presented to the participants. A red ball that fell toward a flipper could be seen on the computer screen during each trial. In order to hit a specific target, participants had to activate TEO with the correct timing (with a timing accuracy of 4 ms) so the flipper could rotate and have the ball to bounce upward toward the target. TEO was activated when the participants flexed their wrists at a torque ≥ 0.5 Nm. One out of three feedback messages were provided during each trial, depending on the participant’s timing accuracy (“Wow! Just on time!,” “Too early! Hit later!,” and “Too late! Hit sooner!”). The pinball simulator was created using LabVIEW^TM^ 2013 software.

### Haptic Guidance and Error Amplification Algorithms

The algorithms were slightly modified from those that we used in a previous study (Milot et al., 2010). To decrease participants’ timing errors during HG, we wanted to delay or speed up the start of the robot when the participants initiated wrist movement too early or too late, respectively. The exact opposite was done to increase errors during EA. More specifically, *t* = 0 was defined as the time in which the ball began to fall toward the flipper. Tbp was defined as the time in which TEO was activated. So:

Tbp=Tip+Dc⁢      (1)

where Tip was the time in which the motor sensors detected the initiation of the participant’s wrist flexion and Dc was defined as the programmed delay in which the participant initiated movement and TEO was commanded to move. The values that ensured success for hitting each target were defined as Tbd and Tid, therefore:

Tbd=Tid+Dcd⁢      (2)

where Tbd was defined as the time in which TEO needed to move in order for the ball to bounce back in time to hit the target, Tid was the desired time in which the participant should have initiated movement, and Dcd was a constant (0.5 s).

Next, Ep was defined as the timing error in which the participant initiated movement, thus:

Ep=Tip−Tid⁢      (3)

Furthermore, Eb represented TEO’s timing error, where:

Eb=Tbp−Tbd=Ep+Dc−Dcd⁢            (4)

We wanted Eb to be proportional to Ep, so:

Eb=kEp⁢         (5)

where *k* was defined as the EA gain. By substituting equation 4 into equation 5 and solving for Dc, we obtained the following equation for the programmed delay:

Dc=Dcd+Ep(k−1)⁢          (6)

Equation 6 was used to establish the delay between when the participant initiated wrist movement, and when TEO began to move, in order to proportionally decrease or increase the participant’s timing errors. Note that no HG or EA trainings were provided when *k* = 1, where a *k* > 1 caused an increase in timing errors and a *k* < 1 resulted in a decrease in timing errors.

Furthermore, knowing that baseline skill level can influence motor learning during HG or EA (Milot et al., 2010), we wanted to adjust each participant’s *k*-value to his own skill level. We did so during a 39-trial baseline adjustment phase (B2), where participants played at a constant game difficulty (*k* = 0.4). When the B2 phase was completed, we classified each participant’s timing errors in an ascending order and chose the 12^th^ *Ep*-value. This 12^th^ *Ep*-value was chosen based on the fact that we wanted subjects to experience at least a 30% rate of success in the subsequent baseline (B3) and retention conditions (RCs). Afterward, taking the upper limit of timing accuracy in order to ensure a successful hit, that is 4 ms (corresponding to Eb), we calculated each participant’s final *k*-value using equation 5.

$$k=4/12^{th}Ep\,\;\;\;\;\left(7\right)$$

The choice of a *k* = 0.4 was driven by our previous study, where the maximum *k*-value reached among the young participants was 0.27. It was reasonable to think that seniors would not reach a *k*-value higher than 0.27 at the end of the B2 adjustment phase. Thus, the robot was providing some help during B2 but to a much lower level than what the subjects really needed to be successful at least 30% of the time, just like during B3 and the RCs.

For each condition, the *k*-value was increased or decreased by 90%, in EA and HG, respectively, to increase and decrease the participant’s timing errors. This 90% change in the *k*-value was sufficient to significantly produce a difference in error between both HG and EA training conditions (Milot et al., 2010).

### Study Design

**FIGURE 2:**
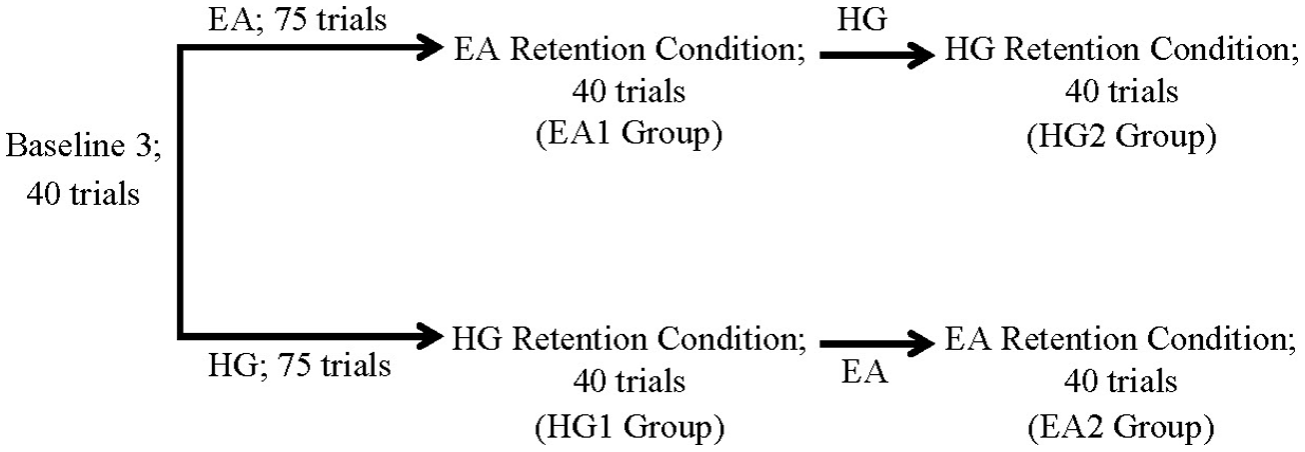
Study design. EA, error amplification; HG, haptic guidance.

Participants were randomly assigned to the two testing conditions; those in Condition 1 experienced the EA training first and the HG second, whereas those in Condition 2 received the HG training first followed by EA (see **Figure 2**).

Before each condition, participants played a Baseline (B1) phase to gain familiarity with the task to be played. B1 was set at a fixed *k*-value of 1, meaning that no HG or EA trainings were provided for 40 trials. Next, a 39-trial adjustment phase (B2) served to determine each participant’s *k*-value. After, participants played a B3 phase according to their *k*-value determined in B2, for 40 trials. Afterward, participants received the training phase (EA or HG), depending on which condition they were in. Each training phase had 75 trials and a 60 s pause after the first 40 trials. During both HG and EA, 20% of the trials were catch trials, meaning that the *k*-value unexpectedly returned to baseline to ensure that the participants would remain watchful throughout the training, especially during HG. A RC equal to B3 followed each training session to allow evaluating the impact of HG or EA robotic training. During HG and EA, three targets (yellow, orange, blue) were presented one at the time to the subjects whereas during B3 and the RC conditions, the two remaining targets were shown along with the three trained targets to assess generalization of the task to untrained targets.

### Statistical Analysis

Subjects’ timing errors were calculated for each trial of each condition. Then, for each subject, two values were computed: (1) mean absolute timing errors and (2) related SDs. Afterward, across subjects, the mean and SD of values #1 and #2 were computed and retained for analysis. The normality of data was assessed using Shapiro–Wilk *W-*test, where non-parametric statistical methods were used for non-normally distributed data that could not be transformed. An independent *t*-test was used to assess if the two groups (EA1 and HG1) were comparable at baseline in regards to age and baseline timing errors during B3. Also, Wilcoxon signed-rank tests were used to evaluate the stability of the learning curve for the timing task (comparison of the mean of the first and last 10 trials of B3 for the entire group of subjects) and introduction to HG and EA training (comparison of the last 10 trials of B3 to the first 10 trials of HG or EA for each training group). Next, a paired *t*-test was used to determine the efficacy of each training type (EA1-B3 and HG1-B3) on the improvement of the timing task, not taking into account the crossover study design, as well as a Wilcoxon signed-rank test to look at the SD of the absolute timing errors. The Hill–Armitage approach (Senn, 2002) to crossover study analysis was used to evaluate: (1) the difference in efficiency between the two types of trainings, by comparing the mean change (EA1 - HG2) of the EA1 group with that of the HG1 group (HG1 - EA2), and (2) whether there was no influence of training order administration, by comparing the mean of the sums (EA1 + HG2) and (HG1 + EA2) between groups. Finally, to evaluate the impact of age on the improvement in timing errors, two approaches were used. First, for each training group, a Pearson product moment correlation was performed to evaluate the relation between age and the change in absolute timing score following HG and EA, respectively. Second, following Senn (2002) procedure, a cross-over difference (mean change in absolute timing errors between the RCs of HG and EA, regardless of treatment order) was calculated and put in relation to age, using a Pearson product moment correlation. One-tailed tests were used and the significance level was set at 0.05. All statistical analyses were performed using IBM SPSS^®^ software version 18.

## Results

### Success Rate, Learning Stability, and Baseline Group Comparison

At B1, where no robotic assistance was provided, the mean success rate of the entire group of subjects reached 2 ± 2%. However, at B3, when the game difficulty was adjusted to each subject’s skill level, the overall mean success rate reached 25 ± 13%; confirming that the adjustment phase worked properly.

Also, when comparing the first and last 10 trials of B3 for the entire group, no difference in the subjects’ absolute timing errors was noted (11.2 ± 5.6 ms vs. 10.1 ± 4.4 ms; *z* = -0.88; *p* = 0.19), meaning that a learning stability of the task has been reached before HG or EA trainings were introduced.

Finally, when comparing HG1 and EA1 training groups, no significant difference was noted in regards to age (68 ± 4 years vs. 68 ± 3 years, *t*(30) = 0.24; *p* = 0.41), and overall mean absolute timing errors at B3 (11.7 ± 4.3 ms vs. 9.8 ± 3.8 ms, *t*(30) = -1.33; *p* = 0.10).

### Introduction to HG and EA

When introduced to HG, a significant decrease in the subjects’ absolute timing errors was noted as compared to the last 10 trials of B3 (10.5 ± 4.8 ms vs. 1.4 ± 0.84 ms, *z* = -3.52; *p* < 0.05). On the contrary, when introduced to EA, a significant increase in the subjects’ absolute timing errors was noted when comparing the last 10 trials of B3 to the first 10 trials of EA (9.8 ± 4.2 ms vs. 18.7 ± 6.5 ms, *z* = -3.52; *p* < 0.05). This means that HG and EA robotic training adequately decreased and increased subjects’ timing errors, respectively.

### Impact of Each Training Type on Timing Errors

**FIGURE 3:**
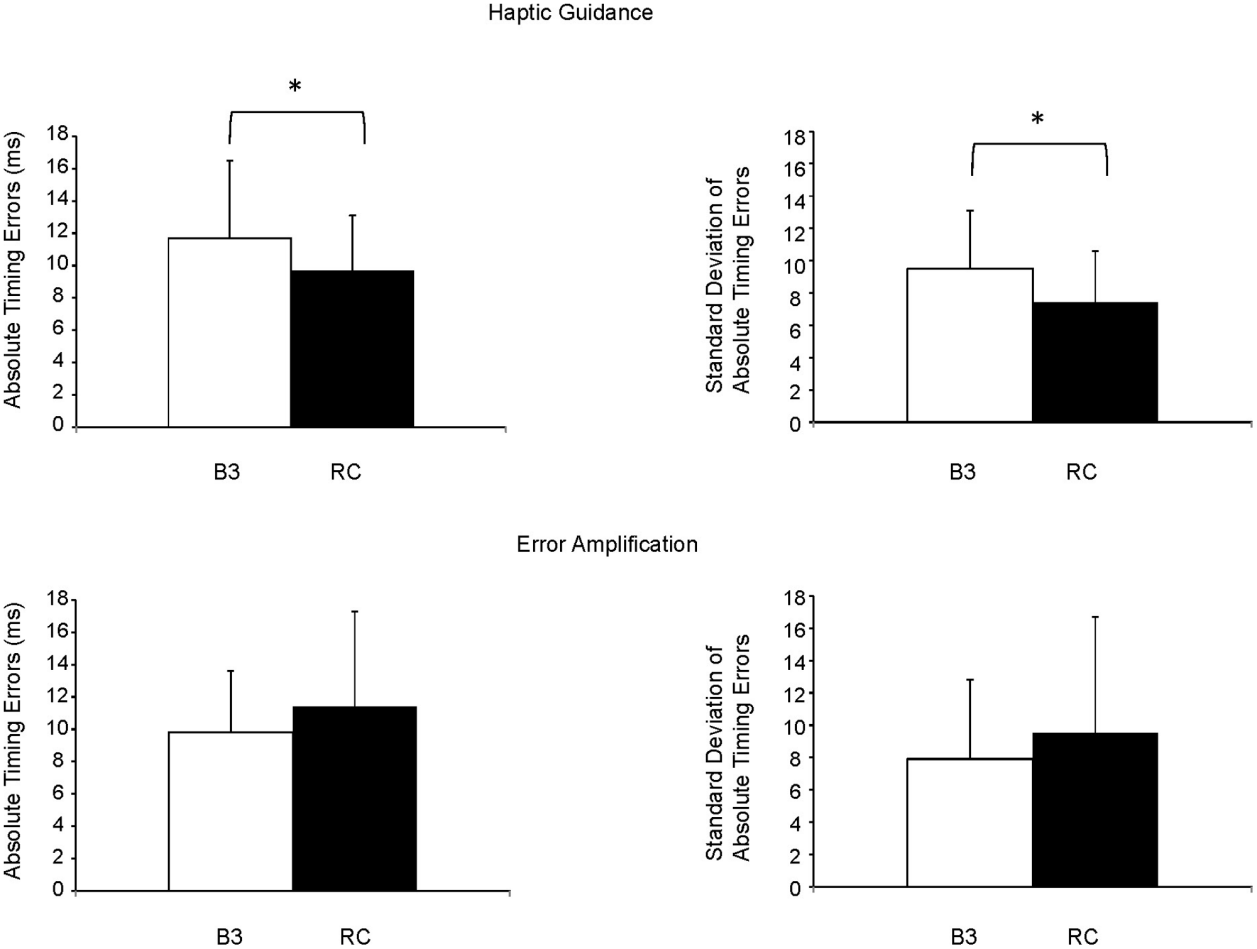
Improvement in absolute timing errors and related SD following HG and EA robotic trainings. B3, baseline 3; RC, retention condition, ^∗^*p* < 0.05.

When comparing the subjects’ baseline performance on trained targets to that of their RC following HG training, a significant decrease in absolute timing errors was noted (11.7 ± 4.4 ms vs. 9.7 ± 3.4 ms, *t*(15) = 1.76; *p* = 0.049). At the same time, subjects were less variable in their timing errors as a significant improvement in the SD of their absolute timing errors was noted when comparing the value of B3 to that of the HG RC (9.5 ± 3.6 ms vs. 7.4 ± 3.2 ms; *z* = -2.17; *p* = 0.01; see **Figure 3**). In addition, when comparing the subjects’ performance on untrained targets between B3 and HG RC, a trend toward a generalization of learning to untrained targets occurred with HG training (11.6 ± 3.2 ms vs. 10.4 ± 3.9 ms; *t*(15) = 1.35; *p* = 0.09). Further analysis also showed that during HG training, the subjects remained alert throughout the training, even though the robot provided them help, since no difference between the absolute timing errors of HG catch trials and B3 was observed (11.0 ± 3.9 ms vs. 11.7 ± 4.4 ms; *t*(15) = 0.68; *p* = 0.25).

Following training with EA, no difference in the subjects’ absolute timing errors on trained targets was observed when comparing timing errors of B3 to that of the EA RC (9.8 ± 3.8 ms vs. 11.4 ± 5.9 ms; *t*(15) = -1.16; *p* = 0.13). In addition, a trend toward a worsening in the variability of the absolute timing errors was observed (7.9 ± 4.9 ms vs. 9.5 ± 7.2 ms; *z* = -1.5; *p* = 0.07; see **Figure 3**) as well as no generalization to untrained targets (10.6 ± 5.1 ms vs. 11.7 ± 5.6 ms; *t*(15) = -1.08; *p* = 0.15). Finally, like HG training, no difference between the subjects baseline performance at B3 and the one during EA catch trials was noted (9.8 ± 0.94 ms vs. 10.7 ± 5.3 ms; *t*(15) = -1.02; *p* = 0.16).

Finally, when comparing the change in absolute timing errors between HG1 and EA1 groups, a significant difference was noted (1.9 ± 4.5 ms vs. -1.6 ± 5.3 ms; *t*(30) = -2.02; *p* = 0.03). In other words, training with HG was more beneficial to learning the timed-based task than training with EA.

### Comparison of the Efficacy of HG and EA Robotic Training on Improvement of Timing Errors and the Impact of Age

When looking at the subjects’ absolute timing error on trained targets, the Hill–Armitage statistical analysis revealed no difference in the efficacy between HG and EA robotic trainings (*U* = 102; *p* = 0.17) and no effect of training order administration on the learning of the timing task (*U* = 109; *p* = 0.25). Although no significant difference in efficiency between the two types of training was noted, when looking at the change in absolute timing errors between RCs of both training groups (HG1/EA2 vs. and EA1/HG2), we performed further analyses by comparing the absolute timing errors of B3 to those of EA2 and HG2. Thus, if looking at the absolute timing errors of B3 to that of EA2, for the group that trained first with HG, a significant improvement in timing errors was noted (11.7 ± 4.4 ms vs. 9.9 ± 4.3 ms; *t*(15) = 2.01; *p* = 0.03). In addition, no difference in absolute timing errors between HG1 and EA2 was noted (9.7 ± 3.4 ms vs. 9.9 ± 4.3 ms; *t*(15) = 0.14; *p* = 0.44). This means that if training first with HG, no worsening in the subjects’ performance occurred after training with EA. On the other hand, when looking at the absolute timing errors between B3 and HG2, for the group that trained first with EA, a trend toward a worsening in timing errors was noted (9.8 ± 3.8 ms vs. 11.7 ± 4.4 ms; *t*(15) = -1.5; *p* = 0.08). In addition, no difference between the absolute timing errors of EA1 and HG2 was observed (11.7 ± 4.4 ms vs. 12.6 ± 7.7ms; *t*(15) = 0.81; *p* = 0.22). This means that HG training was not effective, if given after EA training.

**FIGURE 4:**
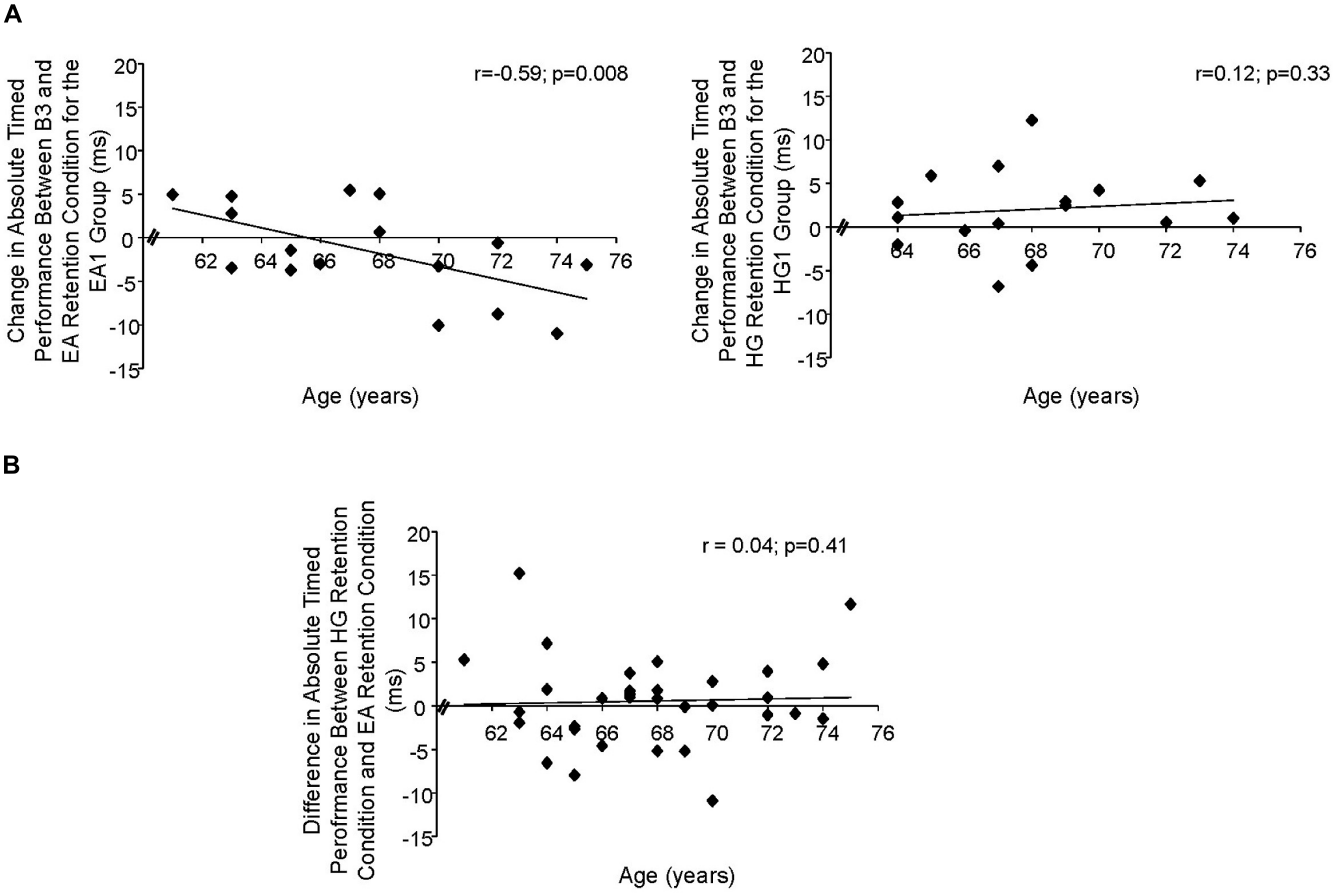
Correlation between age and: **(A)** the change in absolute timed performance for EA1 group and HG1 group. Note that a negative value means a worsening of performance; **(B)** the cross-over difference between the absolute timing errors of the HG RC, and EA RC, *p* < 0.05.

When looking at the impact of age on the subjects’ absolute timing errors for each training group, no significant relation was found for the HG1 group (*r* = 0.12; *p* = 0.33). On the other hand, a significant relationship was obtained for the EA1 group (*r* = -0.59; *p* = 0.008); meaning that for the oldest subjects, training with EA was even more detrimental to learning. In addition, age was not related to the change in subjects’ performance from HG RC to EA RC (*r* = 0.04, *p* = 0.41; see **Figure 4**).

## Discussion

The results of the current study showed that a robotic hand device aimed at reducing healthy seniors timing errors was effective in improving learning a timing-based task, regardless of age. However, improved learning was mostly limited to trained targets. On the other hand, artificially increasing seniors’ timing errors with the use of the robotic device did not promote learning and its generalization. It actually worsened performance with increasing age.

The fact that HG training did improve subjects’ performance further supports the use of HG training for learning time-based tasks (Marchal-Crespo et al., 2010, 2013; Milot et al., 2010; Luttgen and Heuer, 2013). For example, the study of Marchal-Crespo et al. (2010) obtained a significant improvement in steering timing when healthy seniors trained with HG as compared to no guidance. Likewise, in our previous study on healthy young subjects using a similar pinball-like task (Milot et al., 2010), for subjects less-skilled at the task, that is having a *k*-value < 0.1, HG seemed to benefit learning more. Looking at the mean *k*-value of the current subjects (mean *k*-value of 0.06), they did fall within this less-skilled subcategory. As mentioned by Luttgen and Heuer (2013), if a task’s characteristic is difficult to demonstrate either visually or verbally, such as the timing of a task, robotic guidance may provide a helpful role, especially for subjects who struggle with the task.

However, HG training did not translate into generalization of performance, although a trend was noted. Lack of generalization following HG training has been found in several other studies using a variety of tasks (Marchal Crespo and Reinkensmeyer, 2008; Marchal-Crespo et al., 2013). As mentioned by Marchal Crespo and Reinkensmeyer (2008), it could be thought that the current subjects relied too much on the robotic assistance during training with HG, hindering generalization of learning to other targets. Yet, this is not the case as the subjects’ timing errors during HG catch trials did not increase when the robotic assistance was unexpectedly removed as compared to their B3 timing errors. Another explanation could come from the formation of an internal model of the task by the motor system. Indeed, formation of an internal model of the task is important to allow generalization of performance beyond the position (Grafton et al., 2008) or timing (Milot et al., 2010) of trained targets. In young healthy subjects, it was demonstrated that the motor system does indeed create an internal model of timing that can serve for generalization (Milot et al., 2010). However, the creation of an internal model is driven by errors (Thoroughman and Shadmehr, 2000) and because our subjects’ *k*-values were small, they experienced a reduced range of timing errors (Marchal Crespo and Reinkensmeyer, 2008). This might have prevented the motor system to form an adequate internal model of the timing task to allow generalization of performance to untrained targets.

The fact that the subjects’ timing errors decreased after HG training suggest that as one ages, learning can still occur, supporting results of previous studies on seniors’ ability to learn new tasks (van Dijk et al., 2007; Marchal-Crespo et al., 2010; Pietschmann et al., 2011). Knowing that with age, motor performance usually worsens (Hoogendam et al., 2014) it was expected that HG would have been more beneficial for the oldest subjects. However, no relation with age and the change in timing errors following HG was obtained. Further looking at the data, no relation between age and *k*-value was also noted (data not shown); meaning that our oldest subjects did not systematically perform worst at the task to begin with and thus did not necessarily need more help from the robot while playing. To support, Marchal-Crespo et al. (2010) did not find an age dependent relation with motor performance while training with HG on their timing task. Age became an important factor when looking at the long-term retention of the learned task, which was not assessed in the current study.

When looking at the impact of EA training on improvement in timing errors, no significant change in either the absolute timing errors or related SDs was noted. This is not in line with studies on young healthy subjects where EA training translated into a significant improvement in learning various tasks (Patton and Mussa-Ivaldi, 2004; Milot et al., 2010; Marchal-Crespo et al., 2014). Consequently, it seems that exaggerating timing errors in the course of learning for individuals that are more prone to present baseline timing deficits because of normal aging might not be a good strategy to boost learning as for young individuals. Contrarily, following the results of our previous study, for young subjects that were less-skilled at the timing task, EA training did not improve learning (Milot et al., 2010). Explanation of this result was based on the challenge-point theory (Guadagnoli and Lee, 2004), which suggests that learning is a function of both skill level and task difficulty. Thus, for the less-skilled subgroup of young healthy subjects, it was thought that EA training was too challenging for their skill level, overwhelming the motor system with too much information to process and thus hindering any learning. This could be especially true for the current senior subjects knowing that slowness in information processing is observed in the elderly as compared to young individuals due to changes in structural and functional features of the nervous system (Seidler et al., 2010). This is supported also by the significant negative relation found between age and deterioration of timing accuracy following EA training. Thus, with age, EA training could have a more pronounced detrimental effect on learning. On the other hand, it seems that if EA training is given after HG training, no worsening of performance occurs. It is possible that teaching seniors how to perform a timing-task first allows them to improve their skill level and thus makes EA training less challenging.

Although age had a significant negative impact on learning for the EA training group, it did not play an important role when related to the change in score between HG and EA RCs when taking into account all subjects. This could be related to the fact that even though HG training significantly improved subjects’ timing errors, its efficacy was not superior to EA training, when looking at the mean change in absolute timing errors of the RCs between the two groups. The high variability in training responses, often observed with aging (Seidler-Dobrin et al., 1998; Marchal-Crespo et al., 2010), could have prevented the detection of the superiority of HG robotic training over EA when taking into account all subjects, and in parallel precluded to a significant impact of age on the change in score between both types of training.

### Limits

Even though the task that was practiced in this study was similar to the one used in a previous study on young healthy subjects, it was difficult to directly compare the results of both studies since the robots, as well as the determination of each subject’s skill level, differed. The inclusion of a control group with young healthy individuals in the current study would have helped to better interpret the impact of aging on the learning of movement timing. However, when looking at the *k*-value of both studies, representing each subject’s baseline skill level, it is noticeable that the senior subjects did have on average lower *k*-values than young subjects (0.06 vs. 0.15), possibly suggesting a decline in motor timing ability with age. In addition, since no *k*-values reached a value greater than 1 during EA, one could say that no true EA was provided during EA. This fact was also acknowledged in a previous study with young healthy subjects, where it was hypothesized that providing true EA could have been too demanding and thus detrimental to learning. This is even truer with the elderly subjects of this study since, as opposed to young healthy subjects, no learning occurred with EA when the error gains were smaller than 1. Nevertheless, our elderly subjects experienced a significant increase in their timing errors when comparing their baseline performances to their performances when being introduced to EA. Also, one could ask about the relevance of using a pinball-like game to assess timing performance of seniors instead of a more meaningful timing task. The rationale behind the choice of this task was based on the study by Wishart et al. (2000) which suggested that to better evaluate the impact of age on learning, the task should be an unfamiliar one, requiring effortful processing, as opposed to a task that is automatic, involving almost unconscious processing. Thus, by choosing a pinball-like game, we thought that this task was unfamiliar enough to the subjects to properly assess the effect of age on learning. Finally, the long-term retention as well as the clinical importance of a 2-ms improvement in timing following HG training were not established in the current study. Was the improvement in timing maintained over time? Was the change in timing important enough to positively impact seniors’ performance in their everyday activities? Because studies on motor timing in the elderly are scarce, these questions are yet to be answered and more studies are needed to explore these questions. Nevertheless, knowing that the current robotic training significantly and positively helped improve elderly timing, its use with neurologically impaired individuals, where timing deficits can be even more substantial, is worth evaluating. A study is underway to assess the impact of both HG and EA robotic trainings on timing improvement for post-stroke individuals.

## Conclusion

This study evaluated the impact of both HG and EA robotic trainings on the improvement of motor timing in healthy seniors. The results showed that HG was beneficial to learning, with subjects improving their timing accuracy regardless of age. However, learning was restricted to the targets in which practice occurred. No improvement in seniors’ timing errors was noted following EA training. Moreover, a worsening of performance was noted with age after EA training, suggesting that this type of training can be detrimental to learning as one age. Future research should look at the long-term impacts of HG and EA robotic trainings as well as the effects of these robotic trainings on the performance of daily activities to validate their clinical usefulness, particularly with impaired populations.

## Authors Contributions

Conception and design the experiment: MHM, HC

Collection of data: AB

Analysis and interpretation: MHM

Writing of the manuscript: MHM, AB

Revising the manuscript: MHM, AB, HC
